# Levothyroxine Therapy in Gastric Malabsorptive Disorders

**DOI:** 10.3389/fendo.2020.621616

**Published:** 2021-01-28

**Authors:** Camilla Virili, Nunzia Brusca, Silvia Capriello, Marco Centanni

**Affiliations:** ^1^ Department of Medico-Surgical Sciences and Biotechnologies, Sapienza University of Rome, Latina, Italy; ^2^ Department of Experimental Medicine, Sapienza University of Rome, Rome, Italy; ^3^ Endocrinology Unit, Santa Maria Goretti Hospital, AUSL Latina, Latina, Italy

**Keywords:** hypothyroidism, levothyroxine, malabsorption, *Helicobacter pylori*, proton pump inhibitors, gastritis, liquid levothyroxine, softgel levothyroxine

## Abstract

Oral levothyroxine sodium is absorbed in the small intestine, mainly in the jejunum and the ileum being lower the absorption rate at duodenal level. The time interval between the ingestion of oral thyroxine and its appearance in the plasma renders unlike a gastric absorption of the hormone. However, several evidence confirm the key role of the stomach as a prerequisite for an efficient absorption of oral levothyroxine. In the stomach, in fact, occur key steps leading to the dissolution of thyroxine from the solid form, the process bringing the active ingredient from the pharmaceutical preparation to the aqueous solution. In particular, gastric juice pH, volume, viscosity, as well as gastric emptying time seem to be the most important limiting factors. These hypotheses are confirmed by the detection of an increased need for levothyroxine in patients with *Helicobacter pylori* infection, chronic atrophic gastritis, gastroparesis, or in simultaneous treatment with drugs interfering with gastric acidic output. The aim of the present article is to focus on the knowledge of pathophysiologic events that determine the absorptive fate of traditional (tablet) and alternative thyroxine preparations (softgel capsule and liquid solution) in patients bearing gastric disorders.

## Introduction

Levothyroxine sodium monotherapy is usually prescribed as treatment in replacement mode for hypothyroid patients worldwide (1). The need for an individually tailored dose has been strongly suggested (2). However, a significant number of patients fail to show a biochemical and/or clinical response and larger doses of thyroxine are required to reach the target serum TSH concentrations (3). Long-term suboptimal treatments have detrimental effects on body homeostasis (4). Frequent changes of dose and repeated diagnostic procedures in these patients have been related to incremental health costs (5). The causes of an increased thyroxine requirement have been recently reviewed (6). Among these, the role of the altered gastric physiology on the subsequent intestinal T4 absorption has been repeatedly highlighted (7–9). The mechanism by which intestinal absorption of thyroxine is impaired in patients with gastric disorders is unclear but seems to pertain to the chemical and physical properties of both naïve and salificated thyroxine molecule (10). Levothyroxine, the levo-isomer of thyroxine, is insoluble in water and in other usual organic solvents (11). The salification process by a saturating excess of sodium hydroxide leads to the sodium salt production that is the compound used in every pharmaceutical preparation of thyroxine (12). The oral is the preferred route of administration, due to safety and patients’ preference (13). Oral levothyroxine absorption is incomplete with reported percentages of about 70% of the administered dose (14). The actual site of absorption is represented by the jejuno-ileal tract while only a few part of oral thyroxine is absorbed in the duodenum (14). Unlike the rat, no absorption in the large bowel has been described in humans (15). Furthermore, the study of the lag time between thyroxine ingestion and its appearance in the plasma excludes the possibility of absorption occurring in the stomach (15). However, several clinical studies suggested that the variations of gastric physiology might have a deep impact on oral thyroxine bioavailability, leading to an increased need for the drug. We aimed at reviewing the known and unknown on the role of the gastric environment in the absorption of oral thyroxine.

## Gastric Contribution to Drugs Bioavailability

**Figure 1:**
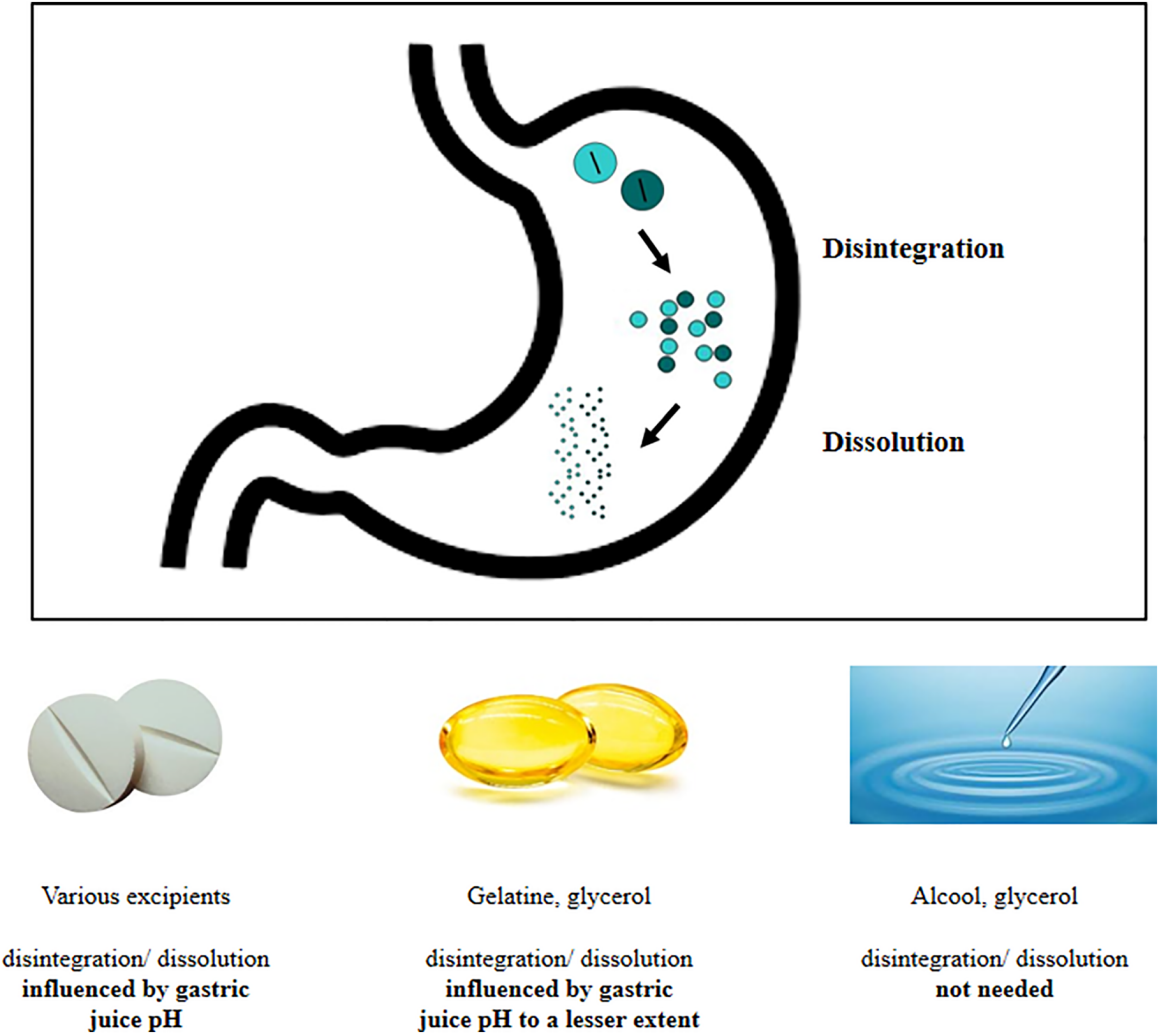
Delivery of active ingredient at gastric level: behavior of different thyroxine formulations.

Most of the drugs are absorbed at intestinal level. This assumption is based on its large surface extension and on the presence of different transporters on mucosal membrane (16). Absorption by duodenal mucosa is in turn regulated by its integrity, motility, mucus composition, and resident microbial population (16). On the contrary, drugs absorption from the stomach is usually thought to be negligible, although a passive diffusion through the gastric wall has been hypothesized and proven for compounds such as ethanol and small neutral molecules (16). The gastric absorption seems to be related to the ionization status of the drug that, in turn, depends on the gastric juice pH: in fact, in an acidic environment, acidic drugs are mainly present and absorbed in a unionized form, being this process negligible for basic drugs (17). However, because of the paucity of papers on this topic further studies are warranted. Anyway, the gastric environment exerts profound effects on drugs behavior and pharmacokinetic. In fact, several steps must be taken into account when analyzing the so-called “gastric phase,” which represents a pivotal prerequisite for the intestinal drug absorption (16). Once reached the stomach, the drug undergoes disintegration, dissolution, and possible precipitation; furthermore, the active ingredient must reach the actual site of absorption. Disintegration causes the release of the active ingredient from the solid form. The duration of this step is highly affected by the type of the formulation and by the excipients used (tablets, capsules, immediate-release formulations), the fasted or fed state, the gastric residence time, and the gastric motor function (16, 18). Simultaneously, the dissolution of the drug occurs. The dissolution process consists in the release of solute molecules from the solid phase to the liquid one, represented by the gastric juice. Again, this process may be affected by physicochemical characteristics of the drug (e.g. particle size and polymorphisms) and by physicochemical conditions (19) on which the role of gastric juice pH and viscosity stand out (20). Several drugs (21), including levothyroxine, share these processes (**Figure 1**).

## Levothyroxine Structure

The shared characteristic of all thyroid hormones is the thyronine nucleus, a diphenyl ether in which the two planar phenyl groups are oriented at an angle of 120 degrees (10). Four iodine substituents at the 3,5,3′ and 5′ positions and the presence of 4′ hydroxyl group in the outer ring characterize thyroxine molecule. Interestingly, the inner ring contains an alanine side chain, which, at physiologic pH, is usually zwitterionic (i.e., net positive charge at the amine group and net negative charge at carboxylic oxygen atoms). Thus, in the thyroxine molecule, three ionizable moieties exist, two acidic (the carboxylic and the phenolic one) and one basic aminic group possessing three distinct pK_a_ (10). It follows that thyroxine may exist in four different ionization status such as zwitterionic, predominant in the range of pH between 2.46 and 6.91 as well as cationic, anionic, and dianionic predominating at more extreme pH (6, 11) depending on environmental pH. The most common pharmaceutical form is the pentahydrated sodium salt of T4 (22). Mondal et al. (23) have shown that almost two polymorphs of levothyroxine do exist. These authors proved the existence of two crystal structures of T4, whose behavior in solution significantly differs being not comparable in different medium pH. The authors hypothesized that these changes in the pH-dependent solubility might affect the oral availability and absorption of this drug (23). The overall aqueous solubility of levothyroxine sodium at 25°C decrease from medium pH 1 to 3, then reaching a nadir level until pH 7, level that correspond to a new increase of T4 solubility (24). The solubility is together with permeability the basis of the Biopharmaceutics Classification System (BCS) (25). Based on the solubility and the permeability rates high or low, drugs are in fact classified into one of four categories of the BCS. This has been proven difficult for levothyroxine sodium since there are sources classifying it as belonging to each of the abovementioned classes (26, 27). Interestingly, also the formation of large aggregates in aqueous media may enable the compound to reach concentration even higher than 15 mg/100 ml (26).

## Interference With Thyroxine Effectiveness Acting at Gastric Level

### Food and Drugs

Several drugs and foods have been proven to interfere with the oral thyroxine absorption [see for rev (6, 28, 29)]. The mechanisms described seem to affect each step of oral and thyroxine absorption and metabolism and are chiefly exerted: a) by changing the gastric pH or adsorbing thyroxine in the stomach; b) by a possible competition with intestinal transporters or adsorbing thyroxine at the intestinal level; c) by affecting thyroxine binding on plasmatic proteins; d) by modulating catabolic thyroxine processes (6, 28). The first two mechanisms are associated with an increased need for thyroxine and are shared by some interfering foods (6). Food itself may represent a gastric hindrance to the bioavailability of drugs (30), including thyroxine (31). In clinical practice, the timing of food intake and the interval before and after thyroxine ingestion seems not negligible for the subsequent intestinal absorption (31, 32).

As mentioned above, the mechanisms of interference affecting oral thyroxine during the gastric passage are substantially the variations of gastric juice pH and the binding of thyroxine in an acidic environment. The antacids represent one of the most prescribed categories of drugs worldwide: the interference with thyroxine bioavailability has been described for proton pump inhibitors and calcium carbonate (28). The effect of proton pump inhibitors (PPI) seems to be related to their role in increasing gastric juice pH that might impact on disintegration and dissolution phases of tablet thyroxine (see for rev ref. 6), although their effect on thyroxine absorption kinetics was denied by other authors (33, 34). However, the net effect of PPI on levothyroxine pharmacokinetic is more complex and partially due to the complex variations of gastrointestinal physiology that may be restricted to the long-lasting use of PPI (i.e. variations in gastric mucus viscosity, gastric and small intestinal bacterial overgrowth) (35). Singh et al. (36) reported that both acute and chronic ingestions of calcium carbonate, as well as different preparations of calcium, are able to reduce the bioavailability of T4. Calcium carbonate showed a specific ability to bind thyroxine *in vitro*: indeed, it appears to bind thyroxine in a dose-dependent manner when medium pH is two; this binding disappears when the medium pH is 7.4, preventing absorption at the intestinal level (36). The negative impact of some nutrients on levothyroxine absorption has been demonstrated since 1977 (37). Most of nutrients (e.g. soy, fiber-enriched alimony and coffee, etc.) (6, 38, 39) specifically bind oral thyroxine at the intestinal level. Interestingly, however, some of them seem to interfere with thyroxine absorption at gastric level like the fruit of papaya. The specific action of papaya seems to act even at gastric level since this fruit causes a significant reduction in histamine-induced acid secretion (40). Milk ingestion seems to interfere with thyroxine absorption for its protein and calcium content as well as for its alkaline pH (41). Noticeably, most of antacid drugs may reduce the acidic exposure of thyroxine in the stomach but they also adsorb the hormone in the upper intestinal tract (6).

### Gastric Disorders and Surgical Procedures

From a clinical standpoint, the association between gastric and thyroid disorders is very frequent (42). An increased need for thyroxine in patients with gastric disorders has been described in patients with *Helicobacter pylori* infection, chronic atrophic gastritis, in those who underwent gastric surgery or bearing gastroparesis. Among these, *Helicobacter pylori* infection is the most important since its prevalence has been estimated worldwide at 48%, despite wide regional discrepancies (Oceania 24% *vs* Africa 79%) (43). From its discovery in 1982 by Warren and Marshall, the role of *Helicobacter pylori* as cause of inflammatory gastritis in more of 90% of the cases has become clear (44). Usually, *Helicobacter pylori* related gastritis initially involves the superficial layer of antrum mucosa of the stomach with an inflammatory mononuclear and plasma cells infiltrate. This phase of infection may feature an increased gastrin level and increased gastric juice acidity as well (45). Depending on cytotoxicity of bacterial strain and gastric environment characteristics, the degree of gastritis may get worsened up to atrophic pangastritis and intestinal metaplasia, determining hypo to achlorhydria (44). A role of *Helicobacter pylori* infection in impairing oral levothyroxine bioavailability was firstly described in 2006 (7). In this report and in the one by Bugdaci (46), the increased need for levothyroxine was reversed following *H. pylori* eradication. This latter paper also highlighted the possibility of iatrogenic thyrotoxicosis, maintaining the previous doses of thyroxine after the removal of infection (46). Undiagnosed or persistent *H. pylori* infection has been also proposed as a trigger for autoimmune atrophic gastritis (47, 48) through a molecular mimicry with epitopes of H_+_/K_+_ATPase, the acid-producing pump of gastric parietal cells (48). In fact, autoimmune chronic gastritis shows a very high degree of corpus and fundus atrophy of the stomach also featuring positive autoantibodies against parietal cells and/or intrinsic factor (49, 50). This pathologic entity is frequently associated with autoimmune thyroid disorders (42, 51), being this association one of the most frequent cases of polyautoimmunity (42, 52). Thyroid and gastric autoimmune disorders are characterized by the action of environmental triggers on genetic predisposing background, leading to the loss of self-tolerance i.e. of the balance between pro- and anti-inflammatory effector cells pathways (52, 53). The co-presence of thyroid and gastric autoimmune disorders features specific immunoregulatory cytokine profiles (54, 55). Autoimmune atrophic gastritis is characterized by achlorhydria and thus by a high oral levothyroxine requirement (7) being maximal in patients bearing the co-presence of gastric atrophy and *Helicobacter pylori* infection (7). The prevalence of autoimmune atrophic gastritis, which is often underdiagnosed, has been estimated as 0.5–5% (51). Achlorhydria is also a feature of laparoscopic sleeve gastrectomy (SG), the most common bariatric procedure performed in the USA (56, 57). The procedure implies the tubulization of the stomach between 50 and 200 cc in volume while the remaining part of the stomach is removed (27). Despite most of the studies examining thyroxine requirement in SG patients described an unchanged or decreased dose of thyroxine needed by patients, the normalization by body weight clearly indicated an increased need for the hormone following this bariatric procedure (56, 57). Patients undergoing bariatric surgery are often advised to use PPIs and micronutrients that may interfere with the absorption of thyroxine; furthermore, their increased need for oral levothyroxine may be warranted by the variations in volume, acidic output, and motility of the remaining part of the stomach (27). These patients, in fact, often show an acceleration of gastric emptying that may impair the disaggregation and dissolution of tablet levothyroxine (58). To note, an increased need for oral levothyroxine has been described in patients with the opposite motility disorder, i.e. gastroparesis (59, 60). However, its frequency is low and estimated in 9/100,000 men and 38/100,000 women (43).

## How to Suspect Gastric Disorders Affecting Levothyroxine Absorption

**Figure 2:**
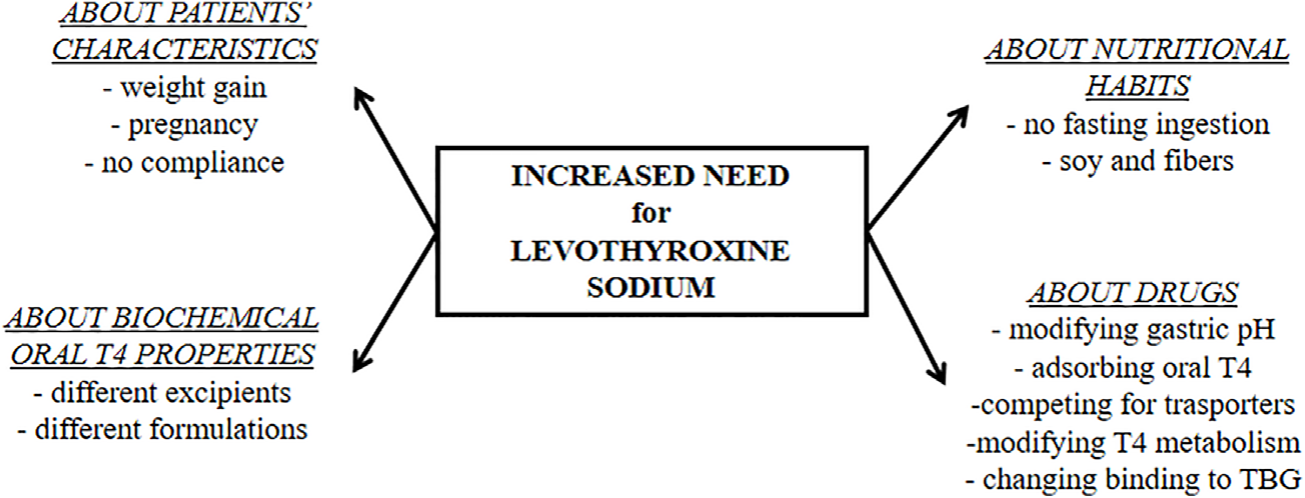
The physiologic and pharmacologic biases of oral levothyroxine treatment that must be excluded before starting a diagnostic workup for malabsorptive disorders.

Three main features may led to suspicion of a gastric disorder: clinical symptoms, malabsorption of drugs and micronutrients, and the presence of a chronic unexplained anemia (6). Despite the narrow therapeutic index, empiric and not targeted doses were widely used without proper characterization for long time (3). On the contrary, an essential prerequisite to detect gastric malabsorption is a careful tailoring of patient’s treatment devoted to find the minimal effective dose of thyroxine (6). Several characteristics of patients and their habits should be evaluated as shown in **Figure 2**. The timing of thyroxine ingestion represents a primary issue to obtain the therapeutic target using the lowest effective dose (7, 31, 32, 61, 62). Other relevant characteristics are the lean body mass or the body mass index, age, reproductive status, and the absence of bias. An accurate pharmacologic anamnestic investigation is, in fact, mandatory to avoid bias from widely used drugs and/or interfering foods (6, 28, 63, 64). Once excluded all these putative biases, a gastrointestinal malabsorption of thyroxine may be suspected (6). The concomitant presence of a macro- or microcytic anemia strengthens the hypothesis of a gastric problem (65, 66). A recent study observed that about half of patients with gastric atrophy presented with anemia that was already severe at the time of diagnosis in one patient out of five (66). Atrophic gastritis is a prevalently silent disease that progresses from a mild chronic gastric inflammation to an advanced stage of atrophy and metaplasia (42). Anemia follows this worsening, proceeding from iron-deficient microcytic phenotype to vitamin B_12_ deficiency-associated macrocytic anemia (pernicious anemia) (65, 66). This latter is a consequence of vitamin B12 malabsorption in turn due to intrinsic factor deficiency (66) whereas the reduced gastric acid secretion lowers iron absorption in iron-deficient anemia (65). Some of these characteristics may prompt a screening for gastrointestinal disorders. The screening for these associated disorders has been recently described and reviewed (6).

At gastric level, the presence of specific antibodies against parietal cell and against *H. pylori* are reliable markers of suspicion as is for fasting gastrin levels. However, the diagnosis of superficial or atrophic gastritis must be based on multiple biopsies and histological examination (42).

## The Use of Novel Formulation in Thyroxine Increased Need Due to Gastric Disorders

The suboptimal efficiency of treatment worldwide (4) prompted industry to develop novel preparations of sodium levothyroxine. Recently, novel formulations of levothyroxine sodium have been introduced: the soft gel capsules and the liquid solution (67). In the softgel capsules, levothyroxine is dissolved in glycerin and surrounded by a gelatin shell while, in the liquid solution, the hormone is dissolved in 95% ethanol and 86% glycerol (**Figure 1**).

A seminal *in vitro* study analyzed the dissolution at different medium pH of two tablet formulations (one brand and one generic) as compared to a softgel capsule (68). The latter performed better at medium pH >3, at which the dissolution curve of the levothyroxine sodium tablet clearly drops (24, 68). A pharmacokinetic study demonstrated that, in healthy subjects and in fasting conditions, softgel capsule formulation is bioequivalent to tablet thyroxine (69).

The dissolution time of the softgel capsule preparation has been directly observed during an endoscopy session in a healthy volunteer, demonstrating that the capsule completely disappeared 21 min following its ingestion (70). Even when analyzed in patients bearing disorders or using drugs increasing gastric juice pH, the softgel formulation performed better than the traditional one (9).

The better performance of softgel formulation in maintaining target TSH levels, despite a lower dose as compared to the tablet one, has been demonstrated in most of the patients bearing superficial gastritis, gastric atrophy, and resistant-to-treatment *Helicobacter pylori* (71). Furthermore, two case reports described patients bearing gastroparesis who benefited from the switching to softgel thyroxine to overcome the refractory hypothyroidism due to gastric motility impairment (72, 73)

The clinical efficacy of softgel formulation in a patient concomitantly treated with proton pump inhibitors has been described in a case-report (74). The better performance of softgel was confirmed by the indices of absorption, evaluated following an acute load with 600 mcg of thyroxine of the two formulations (74). Furthermore, the lesser impact of concomitant breakfast ingestion on softgel capsule preparation performance has been reported (75). To note, a study including patients with gastric disorders demonstrated that the switch from tablet to softgel levothyroxine causes a smaller number of dose adjustments, thus saving health costs (76).

The bioequivalence of the liquid thyroxine preparation to tablet thyroxine has been proven but, oving to the fact that the active ingredient is already dissolved, the time to reach systemic circulation is significantly shorter as compared to both tablet and softgel preparations (77). Some case series reported the usefulness of this formulation in small group of patients with active *H. pylori* infection (78) or bearing atrophic gastritis (79). The liquid T4 formulation has been proven helpful also in the case of concomitant treatment with proton pump inhibitors and several drugs with antacid action (80, 81). A further relevant issue is the effect of food co-ingestion on liquid thyroxine absorption: two papers agreed in defining this formulation less sensitive to the interfering action of food when compared to the traditional one (82, 83). Noticeably, a study on more than 50,000 thyroxine treated patients demonstrated a significant reduction in the number of serum TSH measurements after switching from tablet to liquid formulation (84). These results chiefly pertain to patients using drugs interfering with levothyroxine absorption (84). Liquid formulation has been also proposed in a case of sleeve gastrectomy (85). A recent meta-analysis on studies in which patients on tablet T4 showed suboptimal TSH values indicated that the switch to liquid T4 formulation, at the same daily dose, might help in reaching the target TSH levels (86). A further meta-analysis reported no significant differences in patients without malabsorption but claimed that liquid thyroxine is more efficient than tablet L-T4 in treated patients with malabsorption (87).

## Conclusions

Endocrinologists and physicians should be aware of the role of the stomach on the subsequent intestinal absorption when treating patients with levothyroxine.

## Author Contributions

CV and MC conceived of and designed the study. SC and NB performed the literature search. All authors contributed to the article and approved the submitted version.

## Conflict of Interest

The authors declare that the research was conducted in the absence of any commercial or financial relationships that could be construed as a potential conflict of interest.
